# N of 1 case reports of exceptional responders accrued from pancreatic cancer patients enrolled in first-in-man studies from 2002 through 2012

**DOI:** 10.18632/oncoscience.141

**Published:** 2015-03-14

**Authors:** Ignacio Garrido-Laguna, Danielle Tometich, Nan Hu, Jian Ying, Katherine Geiersbach, Jonathan Whisenant, Kai Wang, Jeffrey S. Ross, Sunil Sharma

**Affiliations:** ^1^ Departments of Internal Medicine (Division of Oncology), Huntsman Cancer Institute and University of Utah School of Medicine, Salt Lake City; ^2^ Center for Investigational Therapeutics, Huntsman Cancer Institute and University of Utah School of Medicine, Salt Lake City; ^3^ Oncological Sciences, Huntsman Cancer Institute and University of Utah School of Medicine, Salt Lake City; ^4^ Department of Pathology at Huntsman Cancer Institute and University of Utah School of Medicine, Salt Lake City; ^5^ Utah Cancer Specialists; ^6^ Foundation Medicine, Cambridge, Massachusetts; ^7^ Department of Pathology and Laboratory Medicine, Albany Medical College, Albany, New York, USA

**Keywords:** N of 1, first-in-man studies, pancreatic cancer, *CRKL* amplification

## Abstract

**Purpose:**

To identify exceptional responders among patients with advanced pancreatic cancer enrolled in first-in-man (FIM) studies.

**Methods:**

A Scopus search identified 66 FIM studies that enrolled at least one patient with advanced pancreatic cancer between 2002-2012. Descriptive statistics were used to summarize categorical variables. We also screened *CRKL* amplifications in the FoundationOne™ pancreatic cancer database.

**Results:**

Most FIM studies included targeted therapies (76 vs. 24%). The most common targeted therapy involved cell cycle inhibitors (24%). Pharmacodynamic analyses were more frequently done in trials with targeted therapies (70 vs. 31%, p=0.006). Response rates were similar. Treatment-related death was 0.5%. Skin, cardiovascular and metabolic grade 3-4 toxicities were more frequent with targeted therapies. Four exceptional responses were identified including a complete response to bosutinib (Src Inhibitor) and partial responses to trametinib (MEK inhibitor) (2 patients) and CHR-3996 (histone deacetylase inhibitor). We found that *CRKL* amplifications, a potential biomarker for Src inhibitors, are present in 1% of PDA.

**Conclusions:**

We retrospectively identified extraordinary responses among patients with advanced PDA enrolled in FIM studies with Src, HDAC and MEK inhibitors. We identified *CRKL* amplifications are present in 1% of PDA and need to be evaluated as predictive biomarker for Src inhibitors.

## INTRODUCTION

Pancreatic ductal adenocarcinoma (PDA) is a devastating disease. It is the fourth most common cause of death by cancer in the United States (US). Approximately 46,420 new cases will be diagnosed in the US in 2014 and 39,540 patients will succumb to the disease.[1] It is estimated that it will become the second deadliest cancer by 2020.[2] The 5-year overall survival rate is less than 5%.[3] Modest improvements in survival have recently been achieved with combinations of cytotoxic agents. For patients with advanced disease and a good performance status, FOLFIRINOX or nab-paclitaxel plus gemcitabine have become the new standard of care.[4, 5] However, most of these patients will progress after 6 months on therapy. Approximately 50% of them will nevertheless be eligible for second-line therapy or enrollment on early clinical trials.[6] While evidence supporting the use of second-line therapy is limited, phase 1 and first-in-man (FIM) trials can be considered for eligible patients. [7-9]

The National Cancer Institute (NCI) has launched an initiative to identify exceptional responders to therapy among patients included in clinical trials with drugs that did not obtain FDA approval due to insufficient activity. [10] The proposed definition for an exceptional responder includes patients with advanced cancer who attain a complete response (CR) to therapy or a partial response (PR) lasting at least 6 months. A tissue acquisition protocol will allow collection of pretreatment tissue samples from these patients to identify genetic aberrations that may predict response to study drug. This research is critical as some of the drugs considered inactive in the past may actually be effective for a small subset of patients provided a biomarker of response is identified. Responses in FIM trials are considered rare events, as patients are often given dose levels below optimal biological doses. A single institution study suggested that in the era of molecularly targeted agents (MTA) patients treated at low doses still benefitted from study drugs.[11] However, a retrospective review of CTEP phase 1 and 2 trials suggests that even with MTA there is a dose-response relation. [12] In general, a response in a FIM trial is considered a remarkable finding and possibly an early indication that an actionable genetic aberration is present in the patient's tumor that renders his disease exquisitely sensitive to the study drug being assessed.

Here, we have reviewed FIM studies published in the decade between 2002 and 2012. The goal of this work is to identify exceptional responders to therapy among patients with advanced pancreatic cancer included in those trials. We found four patients that met the criteria for exceptional responders in FIM. In addition, we found that pharmacodynamic studies are more frequently conducted in FIM studies with targeted therapies. We also found that FIM are safe with a mortality rate similar to that reported previously in a broad analysis of phase 1 trials.[13, 14]

Lastly we report a newly identified *CRKL* amplification in one of our patients with metastatic pancreatic cancer. This genetic aberration has been previously reported in patients with NSCLC. This amplification is present in 1% of patients with pancreatic cancer. Additional preclinical work is needed to test whether this amplification has a role in patient selection for treatment with Src inhibitors in this disease.

## RESULTS

### Characteristics of the Published Trials

Our initial search criteria in Scopus (“phase 1 and solid tumors” between 2002-2012) provided 3,065 results. After reviewing those abstracts, 1,015 phase 1 trials were identified, 115 were FIM studies and 66 of those included patients with advanced pancreatic cancer. The class of drug and characteristics of patients included are summarized in Table 1. The median number of patients enrolled in those studies was 40 (range 16-206). Most patients had good performance status (ECOG PS 0-1) at the time of study entry. Most trials were conducted in the US (N= 43, 65.2%), followed by Europe (N=24, 36.6).

**Table 1:** Summary of first-in-man studies enrolling pancreatic cancer patients and patient demographics (N=66 trials, total number of patients = 3,114)

Variables			
Gender	Male	1,734 (55.7)	
	Female	1,376 (44.3)	
Mean Age (SD)		59.46 (3.22)	
ECOG PS 0-1 (Mean, SD)		95.21% (5.61%)	
Trial location	US Europe Other	40 (61%) 22 (33%) 4 (6%)	
Dose escalation	3 + 3 Accelerated dose titration Other	38 (57%) 25 (38%) 3 (5%)	
FIM	Cytotoxic	16 (24.2%)	
	Targeted	50 (75.8%)	
		Cell cycle inhibitor (Aurora kinase, CdK, PLK, kinesin inhibitors)	16 (24.2%)
		Antiangiogenic	9 (13.6%)
		TKI (MET, EGFR, AKT, src)	5 (7.6%)
		Proapoptotic	4 (6.1%)
		mTOR/PI3K inhib	3 (4.5%)
		MEK/RAF inhib	2 (3.0%)
		Antisense	2(3.0%)
		Inmunotherapy	2(3.0%)
		mAb (HGF, IGF)	2(3.0%)
		Proteosome inhibitor	1(1.5%)
		HDAC inhibitor	1(1.5%)
		Farnesyltransferase inhibitor	1(1.5%)
		Thioredoxin inhibitor	1(1.5%)
		Inhibitor M1 aminopeptidase	1(1.5%)

SD standard deviation

### Dose escalation design and toxicity

The most common dose escalation design was a standard 3+3 (38 out of 66, 57.6% followed by accelerated dose titration (25 of 66, 37.9%) (Table 1).

The incidence of grade 3-4 toxicities per system and drug class (targeted vs. cytotoxic) is summarized in Table 2. No differences in incidence of total grade 3-4 toxicities were found between FIM studies with targeted versus cytotoxic drugs (36.5% vs. 35.9%, p=0.79). Certain grade 3-4 toxicities were more frequent in FIM studies with targeted therapies compared to cytotoxic agents. These included: skin and nail (2.3 vs. 0%, p=0.001), cardiovascular (3.5 vs. 1.3%, p=0.01), metabolic system (5.9 vs. 1.1%, p=0.001). Conversely, infections, hematologic, and gastrointestinal toxicities were more frequent in FIM with cytotoxic agents and this difference was statistically significant.

**Table 2:** Incidence of grade 3-4 toxicity per system

system	All		Drug class				p value
			Targeted therapies		Cytotoxics		
	Total #	%	Total #	%	Total #	%	
Treatment related death	15	0.5	9	0.4	6	1.1	0.01
Hematologic	361	12.0	273	11.1	88	15.9	0.002
Skin and nail	55	1.8	55	2.3	0	0.0	0.001
Gastrointestinal	209	7.0	159	6.5	50	9.0	0.04
Pulmonary	45	1.5	37	1.5	8	1.4	0.89
Cardiovascular	94	3.1	89	3.5	7	1.3	0.01
Metabolic	150	5.0	144	5.9	6	1.1	0.001
Fatigue	112	3.8	94	3.8	18	3.2	0.49
Proteinuria	45	1.5	37	1.5	8	1.5	0.89
Infection	9	0.3	5	0.2	4	0.7	0.04
Neurologic	56	1.9	37	1.6	19	3.4	0.004
Total	1093	36.4	893	36.5	200	35.9	0.79

Dose escalation was stopped due to toxicity in 50 out of 66 trials (75.8%). The proportion of trials in which dose escalation was discontinued due to toxicity was not statistically significantly different when we compared cytotoxic agents versus targeted therapies (81.3% vs. 74.0%, p=0.54). A total of 15 (0.49%) treatment-related deaths were reported among 3,062 patients enrolled in those 66 studies (Table 2). Treatment-related deaths were more frequent in FIM studies with cytotoxic agents compared to targeted therapies (1.1% vs. 0.4%, p=0.01).

### Exceptional responses to therapy

The overall response rate was 2.5%. The difference between response rates in FIM with targeted therapies vs. cytotoxic agents was not statistically significant (2.5% vs. 2.6%, p=0.83).

**Figure 1:**
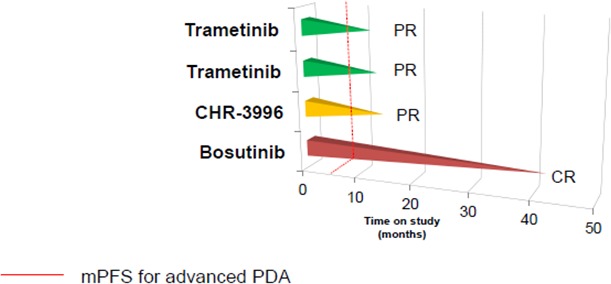
Swim plot with patients with advanced pancreatic cancer enrolled in first-in-man studies who attained exceptional responses to therapy

The NCI proposed definition for extraordinary responses to therapy in patients with advanced cancer includes patients having a complete response (CR) to therapy or a partial response (PR) lasting more than 6 months. Four patients with advanced pancreatic cancer met this criterion in our database. A patient with recurrent pancreatic cancer treated in a FIM study with bosutinib, a Src/Abl tyrosine kinase inhibitor, had an unconfirmed CR and continued to respond for 42+ months after treatment was discontinued.[15] A patient with advanced pancreatic cancer with liver metastases enrolled in a FIM study with CHR-3996, a selective histone deacetylase inhibitor (HDAC), had a PR in the liver lesions and continued treatment for 12 months.[16] Two patients with advanced pancreatic cancer enrolled in a FIM study with trametinib, an oral MEK inhibitor, had confirmed PR to therapy lasting for 10 and 11.7 months.[17] One of the patients treated with trametinib had *KRAS*-positive disease; *KRAS* status was unknown for the second patient. Progression-free survival of these four patients is shown in Figure 1.

### CRKL amplifications in pancreatic ductal adenocarcinoma (PDA)

**Figure 2:**
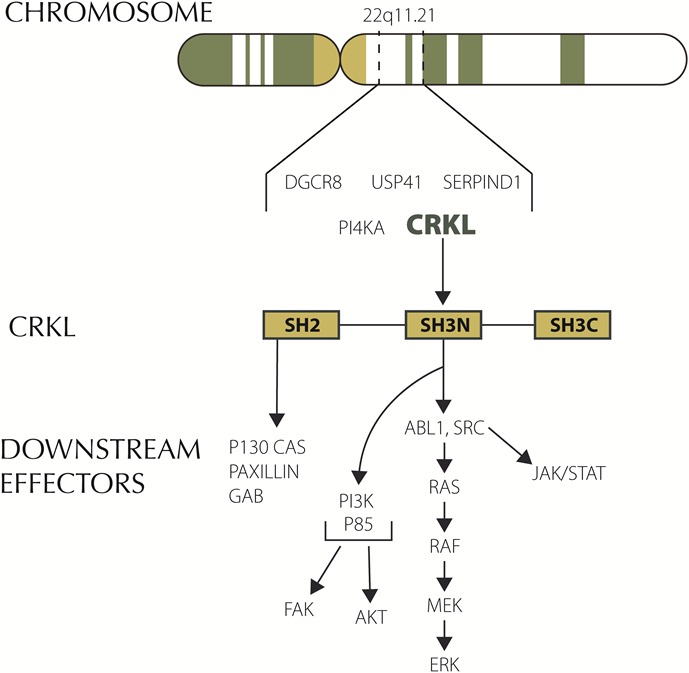
*CRKL* and downstream signaling

We identified amplification in *CRKL* (v-crk avian sarcoma virus CT10 oncogene homolog-like) in one of our patients with metastatic PDA. *CRKL* is located in chromosome 22. It codes for an adaptor protein that unlike other oncogenes (Src, CRK) lacks catalytic activity. CRKL signaling is highly pleiotropic and its role in cancer is still being elucidated. Overexpression of *CRKL* in immortalized human lung epithelial cells promoted epithelial growth factor independent proliferation.[18] Suppression of *CRKL* amplifications in NSCLC that harbor CRKL amplifications induced cell death.[19] Overexpression of *CRKL* in EGFR mutant cells induced resistant to gefitinib. Interestingly comparative genomic hybridization studies have found amplifications spanning 22q11.21 including *CRKL* in pancreatic cancers.[20] These amplifications have not been identified in 905 PDA samples according to COSMIC (data base accessed on October 20th 2014). However, a search in Foundation Medicine database identified 8 (1%) *CRKL* amplifications in 749 patients with advanced PDA. All 8 (100%) of these *CRKL* alterations in PDA were gene amplifications. In an expanded analysis of *CRKL* alterations across all tumor types, 99.5% were *CRKL* amplifications and 0.5% were *CRKL* base substitutions. *CRKL* amplified PDA seems to comprise a unique subset as shown by the paucity of *KRAS* mutations in these patients in our data. 4 out of 8 patients with *CRKL* amplification had *KRAS* mutations while 622 out of 741 CRKL wild-type patients had a mutation in *KRAS* (Fisher's exact test p<0.03). Genomic alterations in *CRKL* appear to be predictive of response to Src inhibitors in preclinical studies with gastric and NSCLC cell lines (Figure 2).[21] Of note, chronic myeloid leukemia (CML) cell lines have the highest levels of *CRKL* mRNA expression according to Cancer Cell Line Encyclopedia (data base accessed on October 20th 2014). Dasatinib, a Src inhibitor is approved by the FDA for the treatment of CML. We identified *CRKL* amplification in one of our patients with advanced pancreatic cancer. He had previously progressed to FOLFIRINOX and nab-paclitaxel plus gemcitabine. He was started on dasatinib, unfortunately he had rapid progression of disease and dasatinib was discontinued after 3 weeks due to declining performance status. Further preclinical work in this disease will elucidate if *CRKL* has a role as a biomarker to identify patients with PDA who may benefit from enrollment in clinical trials with Src inhibitor.

## DISCUSSION

Under the “one-size-fits-all” paradigm patient subsets were often ignored. This precluded drugs targeting genetic aberrations with a low prevalence to show activity in large randomized trials of unselected populations.[22] The model essentially set those drugs up for failure despite any activity in small subsets of patients. Conversely, it was also possible for drugs to gain regulatory approval on the basis of modest clinical benefit despite the potential to be detrimental in specific patient subsets.[23] More recently, a shift in the drug development paradigm toward precision medicine has led to a plethora of biomarker-driven trials that are better positioned to tackle the complexity of individual cancers.[24] In addition, this strategy has proven successful to significantly shorten the time required for drug development.[25, 26] Biomarker-driven trials are critical for efficaciously addressing the complexity of cancer heterogeneity, accelerating the approval of new drugs and ultimately fulfilling the promise of precision medicine.[27] Melanoma has emerged as a major tumor type demonstrating the utility of this strategy in solid tumors with up to five new drugs gaining regulatory approval for this disease in the last five years. However, our inability to completely understand molecular responses to therapy may have led to the halting of clinical development of active compounds in the past. Some patients treated with novel agents attained remarkable responses to therapy yet those drugs frequently never made it through regulatory approval and into the clinic. It is critical to understand responses to therapy at the molecular level in these patients. The NCI is currently leading efforts to identify extraordinary responses to therapy in clinical trials. To this end, the NCI has proposed a broad definition of extraordinary response to therapy. This definition includes patients with advanced cancer who have either a CR or a PR lasting more than 6 months.

In this report, we have analyzed a publically available database of patients with advanced pancreatic cancer enrolled in FIM trials in the last decade. The toxicity and adverse event-related death rates and response rates in these trials are consistent with that previously reported in broader retrospective analysis of phase 1 trials. [13, 14] We also found that although the overall toxicity rate was not significantly different between cytotoxic agent and targeted therapy trials, the hematologic toxicities were more frequently seen with cytotoxics while metabolic, skin nail and cardiovascular toxicities were more frequent in FIM with targeted therapies.

Extraordinary responses to therapy in FIM are rare for several reasons. First, most of these patients have been heavily pretreated. Second, the goal of these trials is to understand drug toxicity and to define a safe dose of the study drug. Patients are often treated at doses that may not be biologically relevant. Overall, however, these trials provide us an extraordinary opportunity to identify patients exquisitely sensitive to therapy.

Our search identified 4 patients with advanced pancreatic cancer treated in FIM studies published in the last decade that met the NCI definition for extraordinary responses to therapy. One of these patients achieved a CR to bosutinib, a Src inhibitor. Src is a non-receptor tyrosine kinase frequently overexpressed in pancreatic cancer. Several Src inhibitors are currently under clinical development. Dasatinib failed to show any significant activity in a phase 2 trial in non-selected patients with advanced pancreatic cancer.[28] Preclinical studies using patient-derived xenografts (PDX) identified high expression of caveolin-1 in PDX sensitive to bosutinib. In addition, a k-TSP classifier including 6 genes was able to predict responses to bosutinib in pancreatic PDX.[29] Lastly, we recently identified through comprehensive genomic profiling *CRKL* amplification in one of our own patients with metastatic pancreatic cancer. *CRKL* is located in the 22q11.21 region. Deletions in this region are typical in patients with DiGeorge syndrome.[30] The role of *CRKL* in oncogenesis has been elucidated in recent years.[31] *CRKL* amplification is the most common mechanism leading to pathway dysregulation and it has been identified in different tumor types. Loss of the tumor suppressor miR-126 which targets Crk has also been described. *CRKL* encodes an adaptor protein with src homology domains that recruits different downstream effectors including JAK/STAT, MAPK, FAK and PI3K/AKT. *CRKL* amplifications had previously been described in pancreatic cancer.[20] Genetic aberrations involving *CRKL* are not reported in COSMIC. However, our data show that 1% of patients with advanced pancreatic ductal adenocarcinoma have aberrations in this gene. An interesting finding was that despite *KRAS* mutations are nearly universally present in PDA, they are only found in 50% of PDA with *CRKL* amplification. This suggests that *CRKL* amplified PDA is a unique subset within this disease. *CRKL* encodes an adapator protein with Src homology domains. Consequently, Src inhibitors may have a role in patients with amplification of *CRKL*.[18, 21] Unfortunately, we were not able to obtain tissue from the institution that treated the patient who achieved a complete response to bosutinib to test for the presence of *CRKL* aberrations. In our N of 1 experience, dasatinib failed to help a patient with *CRKL* amplified pancreatic cancer. Treatment was started 16 months after he had been diagnosed with metastatic disease to the liver. It is possible that *CRKL* is not a predictive biomarker or conversely targeted therapies possibly will be more effective in earlier phases of the disease.[32]

Three patients had a partial response lasting over 6 months. Two patients were treated in a FIM study with trametinib, an oral MEK inhibitor. After attaining a PR, these patients remained on treatment for 10 and 11.7 months. One of them had *KRAS*-positive pancreatic cancer. *KRAS* is mutated in >95% of patients with pancreatic cancer. Activating mutations in the MAPK pathway, including *KRAS,* could predict response to MEK inhibitors. In non-selected patients with advanced pancreatic cancer, a randomized, double-blind, placebo-controlled trial of trametinib in combination with gemcitabine recently failed to improve survival.[33] Lastly, a patient had a PR in a FIM with CHR-3996, a HDAC inhibitor. The patient was able to continue treatment with CHR-3996 for 12 months. Histone deacetylases are components of core signaling pathways in pancreatic cancer such as the cell cycle and notch pathway.[34]

Our study has limitations. We have identified these exceptional responders from published clinical trials. Unfortunately, most patients are not enrolled in trials and, in the case of advanced pancreatic cancer, fewer than 5% of patients are enrolled in clinical trials.[2] Therefore, other, possibly many, extraordinary responses to therapy are likely not included here. Furthermore, we included only published trials. However, we would expect that trials in which responses have been identified are more likely to be published than not. Therefore, we expect publication bias to have little impact in our results. Moreover, we collected extensive information from each FIM, usually not available from abstract presentations.

The next step is to understand these responses at the molecular level. To this end, initiatives to obtain tissue from extraordinary responders to therapy are urgently needed. This initiative may lead to the identification of genetic aberrations that could predict response to targeted therapies and open new venues for treatment of this lethal disease.[35, 36] This could expedite identification of predictive biomarkers and patient subsets more likely to respond to therapy. We envision some challenges with this approach. First, most of these patients may have succumbed to their disease so that only archived tissue will be available. Second, tissue acquisition in patients with pancreatic cancer may be complex as these patients are often diagnosed with fine needle aspirations; however, to confirm that tumoral heterogeneity would not be a clinical problem when biopsy materials were sequenced, published studies using sensitive hybrid capture bases sequencing technology have confirmed that diagnostic PDA needle biopsies/FNA find the same genomic alteration signatures that are found in the tumors when repeat sequencing is performed on the Whipple resections.[37] Lastly, it is likely that we will identify multiple genetic aberrations in these patients. The challenge will be to understand which ones are driving responses to therapy.

## MATERIALS AND METHODS

We conducted an electronic search in the Scopus abstract and citation database using the terms, “phase 1 and solid tumors”. Our search conducted on January 3, 2013 provided 3,065 results. A phase 1 clinical researcher (IGL) reviewed the abstracts and identified FIM studies enrolling at least one patient with advanced pancreatic cancer. Data collection was conducted by two reviewers (IGL and DT) and included: author, journal, year of publication, study drug, mechanism of action of study drug, class of drugs (cytotoxic agent vs. targeted therapy), route of administration, number of study sites, escalation design, dose level treated, number of patients enrolled, median age, gender percentage, ECOG PS, brain metastases allowed or not, grade 3-4 toxicities by system, dose-limiting toxicities, reason for stopping dose escalation, maximum tolerated dose identified versus not, treatment-related deaths, responses and response criteria, pharmacodynamic analysis, expansion cohort, trial location, tumor types included.

In addition, we performed next generation sequencing in one of our patients with advanced pancreatic cancer using two platforms. We screened for mutations in a panel of 48 genes using Ion Torrent. We also performed comprehensive genomic profiling (CGP) for hundreds of known cancer genes using the FoundationOne™ next generation hybrid capture-based sequencing assays. [38] These results were expanded to include a database analysis of *CRKL* alterations in a series of 749 PDA archived at Foundation Medicine, Cambridge, MA. *CRKL* amplification was defined as *CRKL*/CEP22 ratio greater than 2.0.

### Statistical Analysis

Descriptive statistics were summarized as frequency (%) for categorical variables (such as gender) and mean (SD) for continuous variables (such as median age for trials). Toxicity rate data were summarized by system and drug class (targeted therapies versus cytotoxic agents). Response rate, toxicity rate, death rate and stop escalation rate were summarized for both targeted therapies and cytotoxic drug classes. Comparisons of toxicity incidence, response rate, treatment-related death rate between targeted therapies and cytotoxic agents were assessed using the proportion test assuming large-sample results. The statistical analyses were conducted using STATA software (Stata Inc, College Station, TX, USA) version 11 and SAS (SAS Institute Inc., Cary NC, USA) version 9.3 (NH and JY). All statistical tests were performed as two-sided at a 0.05 test level.
